# Development and characterization of an EMS-mutagenized wheat population and identification of salt-tolerant wheat lines

**DOI:** 10.1186/s12870-019-2137-8

**Published:** 2020-01-13

**Authors:** Johanna Lethin, Shahriar S. M. Shakil, Sameer Hassan, Nick Sirijovski, Mats Töpel, Olof Olsson, Henrik Aronsson

**Affiliations:** 1 Department of Biological and Environmental Sciences, University of Gothenburg, Box 461, SE-405 30 Gothenburg, Sweden; 2 Department of Pure and Applied Biochemistry, University of Lund, Box 124, SE-221 00 Lund, Sweden; 3 Department of Marine Sciences, University of Gothenburg, Box 461, SE-405 30 Gothenburg, Sweden

**Keywords:** Bangladesh, EMS, Mutant frequency, Mutagenized population, Salt, Screening, Tolerance, Wheat

## Abstract

**Background:**

*Triticum aestivum* (wheat) is one of the world’s oldest crops and has been used for >8000 years as a food crop in North Africa, West Asia and Europe. Today, wheat is one of the most important sources of grain for humans, and is cultivated on greater areas of land than any other crop. As the human population increases and soil salinity becomes more prevalent, there is increased pressure on wheat breeders to develop salt-tolerant varieties in order to meet growing demands for yield and grain quality. Here we developed a mutant wheat population using the moderately salt-tolerant Bangladeshi variety BARI Gom-25, with the primary goal of further increasing salt tolerance.

**Results:**

After titrating the optimal ethyl methanesulfonate (EMS) concentration, ca 30,000 seeds were treated with 1% EMS, and 1676 lines, all originating from single seeds, survived through the first four generations. Most mutagenized lines showed a similar phenotype to BARI Gom-25, although visual differences such as dwarfing, giant plants, early and late flowering and altered leaf morphology were seen in some lines. By developing an assay for salt tolerance, and by screening the mutagenized population, we identified 70 lines exhibiting increased salt tolerance. The selected lines typically showed a 70% germination rate on filter paper soaked in 200 mM NaCl, compared to 0–30% for BARI Gom-25. From two of the salt-tolerant OlsAro lines (OA42 and OA70), genomic DNA was sequenced to 15x times coverage. A comparative analysis against the BARI Gom-25 genomic sequence identified a total of 683,201 (OA42), and 768,954 (OA70) SNPs distributed throughout the three sub-genomes (A, B and D). The mutation frequency was determined to be approximately one per 20,000 bp. All the 70 selected salt-tolerant lines were tested for root growth in the laboratory, and under saline field conditions in Bangladesh. The results showed that all the lines selected for tolerance showed a better salt tolerance phenotype than both BARI Gom-25 and other local wheat varieties tested.

**Conclusion:**

The mutant wheat population developed here will be a valuable resource in the development of novel salt-tolerant varieties for the benefit of saline farming.

## Background

Wheat is the world’s second most important crop, with a harvest of ca 735 million metric tons during 2018 (www.fao.org). Only corn produces more, at ca 1000 million metric tons. Wheat is grown for its high yield and nutritional benefits, in particular protein, carbohydrates, fiber, fats, minerals and B-group vitamins [1]. It is also the major ingredient in staple foods such as pasta, biscuits, bread, and confectionery products used worldwide and constitutes 21% of the total cereal-based production by the food industry [1, 2]. The production of wheat has steadily increased each year up until 2017 and there was forecast to be a harvest of ca 757 million metric tonnes in 2018. However, for the first time ever, in 2018 the total wheat yield did not deliver the previously predicted increase, mainly due to climate issues (www.fao.org). The human population continues to increase, with cities steadily growing and expanding into rural areas, while the area of arable land decreases due to urbanization and soil destruction. Wheat yield needs to be increased by 70% within the next 40 years in order to meet global demands (www.fao.org) [2, 3].

Salinity is one of the major abiotic stressors affecting crop plants worldwide. Six percent of the world’s arable land and 20% of the world’s irrigated arable land can no longer be used for crop cultivation due to high salinity. In addition, it is estimated that 2000 ha of arable land are lost every day due to salt contamination [4–6].

Crop yields are seriously damaged by salt stress since high salt concentrations strongly inhibit both seed germination and vegetative growth [7]. Excess salt levels cause ionic imbalance within cells, resulting in various degrees of oxidative stress [8]. The ion imbalance occurs as a consequence of excessive uptake of Na^+^ and Cl^−^ ions through the roots, resulting in an accumulation of these ions to toxic levels. This in turn leads to a reduction in essential mineral nutrients like e.g. K^+^ and Ca^+^, negatively affecting plant growth [9]. Moreover, as salt stress especially inhibits developing plant tissues, photosynthesis is reduced due to impaired leaf development, resulting in thicker and smaller leaves [10, 11]. Another negative effect of salinity is the drought stress that occurs when the salt concentration in the soil exceeds the levels inside the root cells, which leads to the withdrawal of water from the root cells due to osmosis. On the other hand, exposure of growing plants to sub-lethal salt concentrations increases their tolerance to more severe salt stress in a phenomenon referred to as acclimation. During the acclimation process the plant differentially regulates a number of stress responsive genes, resulting in various tissues becoming adjusted to Na^+^ and Cl^−^, as well as other ions. One adjustment mechanism is the accumulation of compatible solutes in the cytoplasm; these hinder the entry of harmful ions accumulated in the vacuole into the cytoplasm [12, 13]. Most of the differentially regulated salt stress response genes are up-regulated, but there are also examples of down-regulated genes [14]. Several plant hormones such as auxins, brassinosteroids, gibberellins, cytokinins and abscisic acid are involved in responses to different abiotic stresses, including salt stress [15, 16]. Halophytic plants, i.e. plants that have the ability to grow and survive in high saline soil by restricting root ion uptake, have been invaluable resources when studying salt tolerance [17].

In Bangladesh, the groundwater salt content ranges between 1 and 10 dS/m depending on the region and time of measurement [18]. A major reason for this high groundwater salinity is the Tidal channel, since the water in this channel is brackish, being a mixture of seawater from the Bay of Bengal and freshwater from periods of heavy rain. Thus, the severity of Tidal flooding during the wet season (June until October) will directly be reflected in saline soils during the dry season [19]. As a consequence, large areas of the lands remain fallow in the dry season (November–May) due to excessive salt concentrations in the soil. It is estimated that around 1 million hectares of land in the coastal areas are affected by salt to varying degrees [19, 20]. Ideally, salt-tolerant wheat could be grown in these areas during the somewhat cooler winter season.

To develop wheat varieties with specialized characteristics, e.g. higher nutritional value or increased abiotic stress tolerance, a deeper molecular understanding of these processes and a higher level of variation in current wheat populations would be an advantage. Several efforts have been made during the last few decades to develop salt-tolerant wheat varieties, but with only limited success. In addition, the molecular mechanisms of salt tolerance are still far from being understood [21]. In previous studies two main molecular genetic approaches have been used: marker assisted breeding (MAS) and transgenic breeding [22]. The use of MAS is advantageous since a specific trait in a population can be targeted and bred into elite varieties faster and with greater precision than can be achieved using conventional phenotypic selection methods [23]. Ideally, MAS markers should be developed for those EMS mutations that give rise to desired phenotypes. This would allow efficient crossing of a saline-tolerant phenotype with different elite wheat varieties and at the same time eliminate non-beneficial mutations in the selected mutated lines used in the crossings.

Here we developed an EMS mutagenized population of the Bangladeshi wheat variety BARI Gom-25, which is a hexaploid bread wheat variety containing three genomes (AABBDD), each with seven chromosomes. BARI Gom-25 was released in 2010, and it is semi-dwarf, early maturing and high yielding. It has a 1000 grain weight of 54–58 g, and the crop duration is 102–110 days, plant height being 95–100 cm and panicle initiation time being 57–61 days. Moreover, it is classified as moderately tolerant of salt (~ 8–10 dS/m) and heat. EMS mutagenesis causes random point mutations mainly in the form of G/C to A/T transversions. Mutations will occur in both non-coding and coding regions. The mutations can lead to up- or down-regulation of gene expression, alteration of mRNA stability or changes in protein structure by introducing amino acid changes, stop codons or more rarely frame shift mutations [24]. EMS mutagenesis has been successfully applied to several crops, e.g. oat [25], barley [26], maize [27], and wheat [28], as summarized by Sikora et al., [29]. By inducing a high frequency of mutations in a large population of seeds, and stabilizing the mutations through a number of self-crosses, mutated lines can be developed. Such populations can be screened either for desired phenotypes, e.g. salt tolerance, or directly for mutations in genes of interest. We developed and used a mutagenized BARI Gom-25 population to screen and identify 70 individual lines with a level of salt tolerance high enough to allow them to germinate at 200 mM NaCl. Ultimately, such lines can be developed into salt-tolerant varieties that can be cultivated on saline soils. This would not only be of economic advantage, since an extra growth period can be added in countries like Bangladesh [30], but such crops could also re-utilize and remediate saline land in the drive for more sustainable agriculture.

## Results

### Development of a mutant wheat population

**Fig. 1:**
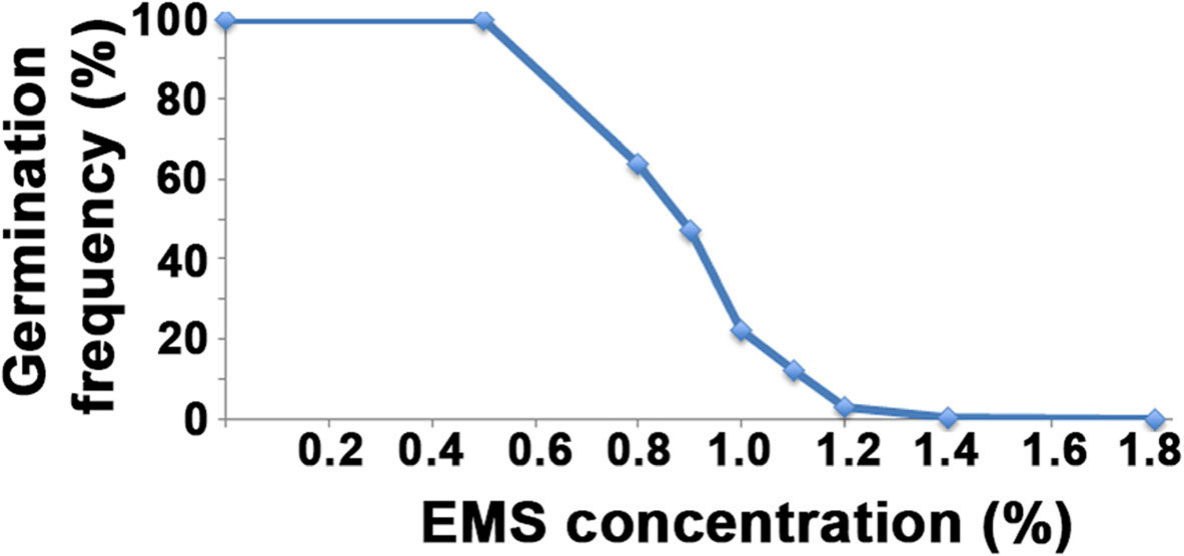
Optimization of EMS treatment of BARI Gom-25. BARI Gom-25 seeds were treated with different concentrations of EMS and grown in a greenhouse under optimized growth conditions for 15 days according to a previous protocol (Chawade et al., [25]). To generate the M1 mutant population, 1% EMS was selected and 30,000 BARI Gom-25 seeds were treated. Out of these, ca 6000 seeds (20%) germinated

**Fig. 2:**
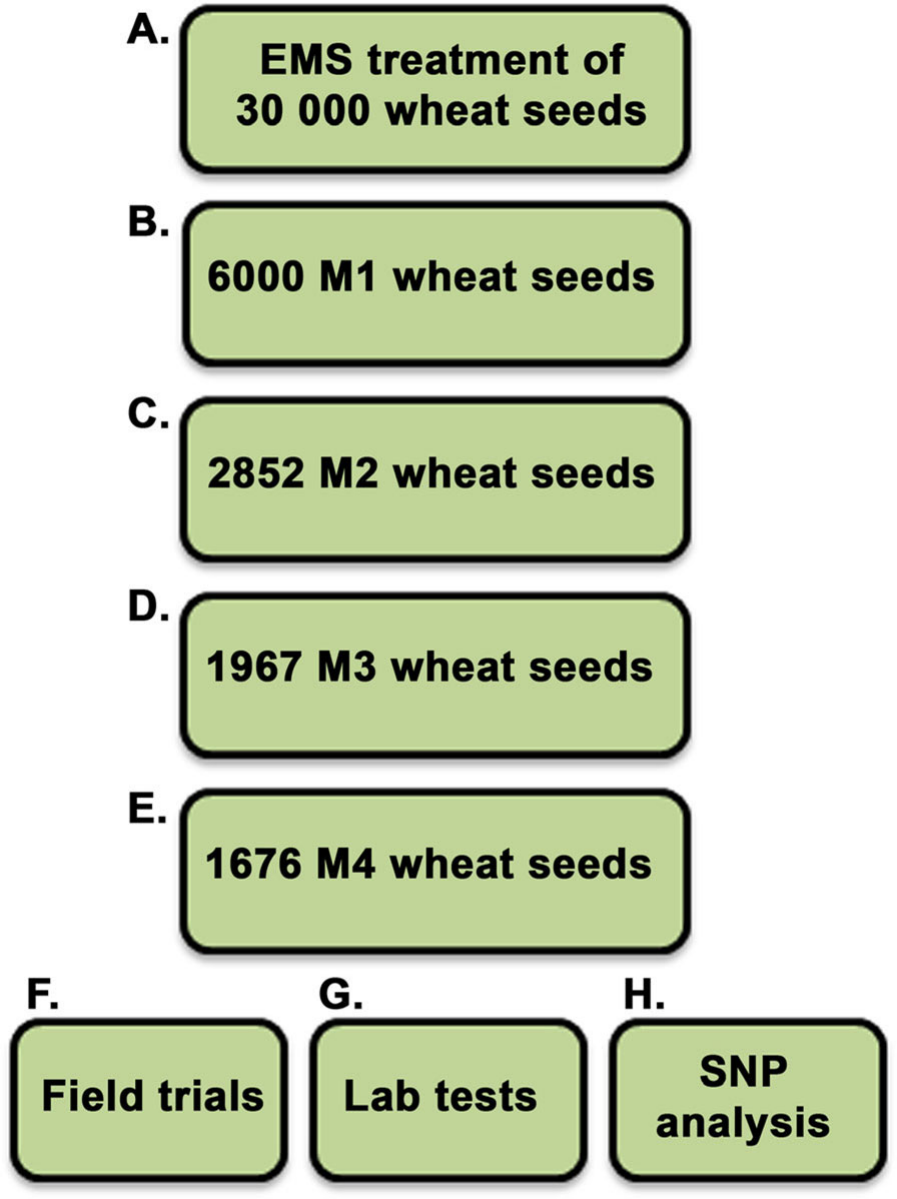
Development of the mutagenized wheat population. Schematic illustration of the steps followed to create, propagate (**a**-**e**) and test (**f**-**g**) the different individual mutagenized lines. M1-M4, mutagenized generation number

**Fig. 3:**
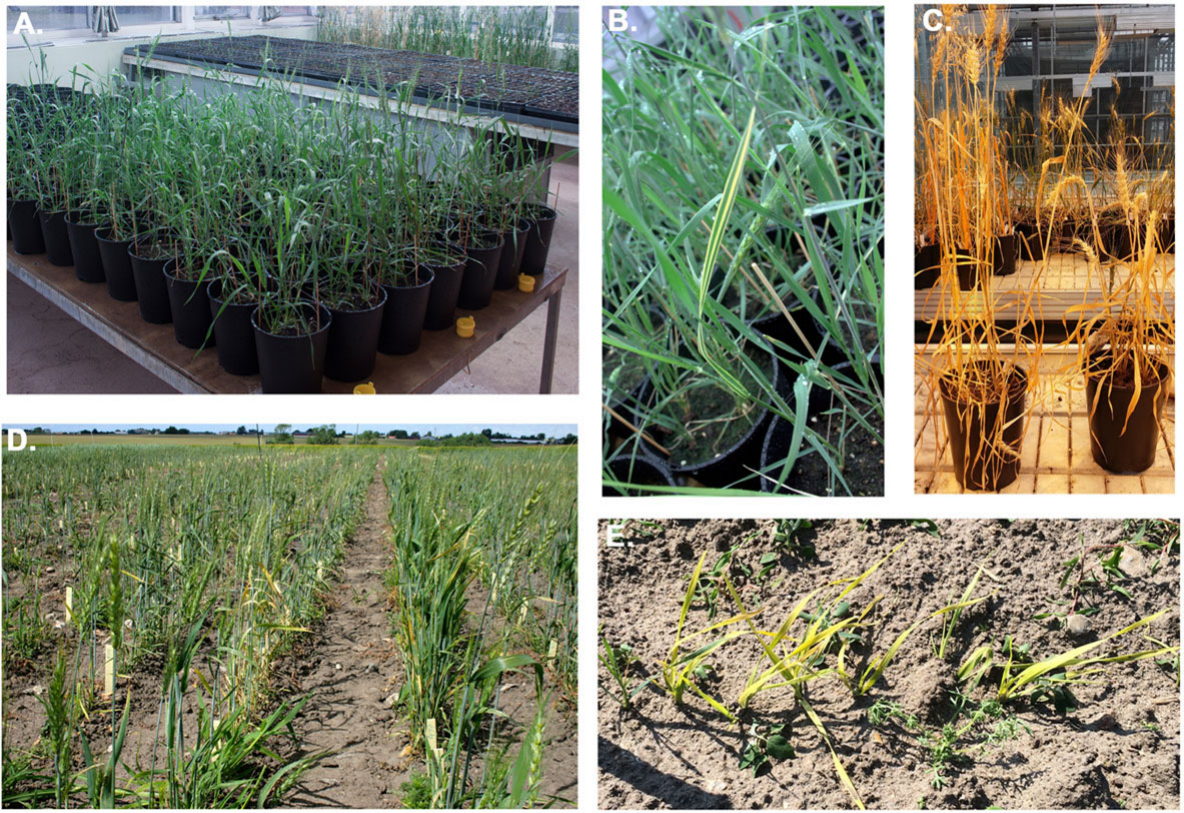
Propagation of the mutagenized wheat population. **a** Large-scale production of the M1 generation in the greenhouse at Lund University. **b** chlorotic and **c** morphological mutant (short height phenotype on the right) observed under greenhouse propagation conditions. **d** Large-scale production of the M3 generation at Borgeby, Sweden with an **e** chlorotic mutant observed in the field

To determine the amount of EMS needed to achieve the highest mutation rate without killing all the seeds due to the toxicity of the compound, an EMS titration curve was established (Fig. 1). In the titration, different concentrations of EMS ranging from 0 to 1.8% were used. From each treatment, 200 seeds were planted. Based on previous studies using hexaploid oat [25] the target was a germination survival rate of approximately 20%. In the wheat system we found that 1% EMS would be optimal (Fig. 1). Since we aimed to have all genes mutated across the whole population, we used 30,000 seeds as starting material for the EMS treatment. From these, approximately 6000 seeds germinated in the M1 generation. One M1 seed from each line was planted out and from these seeds 2852 different lines survived and M2 seeds could be harvested (Fig. 2). These lines were then propagated until the 4th generation, by which 1676 lines still remained. From this population, salt-tolerant lines were identified (Fig. 3). Lines not surviving up to the M4 generation were lost due to sterility, failure to germinate, axes not being filled, increased sensitivity to various diseases, plant death, failure to develop new seeds, etc.

### Confirmation of salt tolerance of BARI Gom-25

**Fig. 4:**
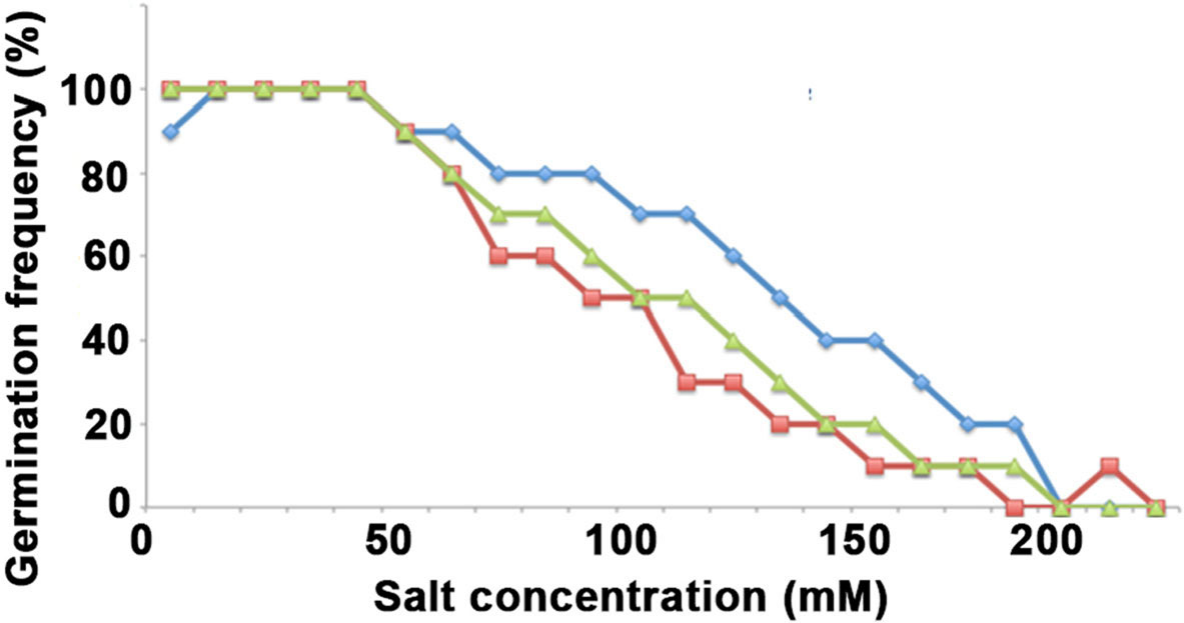
Salt tolerance test on BARI Gom-25 using three different salts. Seeds were germinated at different concentrations of NaCl (blue), MgCl_2_ (red) and KCl (green) for 7 days and the germination frequencies at the different salt concentrations were recorded

To confirm the salt tolerance of BARI Gom-25, as well as to titrate the optimal salt concentration in order to identify more strongly salt-tolerant lines within the wheat mutant population, an initial test was performed with BARI Gom-25 using three different salts and a range of different salt concentrations (Fig. 4). This showed that at ca 100 mM (approximately 10 dS/m) BARI Gom-25 had a germination rate of 80% with KCl and MgCl_2_ and 90% with NaCl, confirming the moderate salt tolerance of BARI-Gom-25 (Fig. 4). At increased salt concentrations, the germination rate decreased and at 200 mM (approximately 20 dS/m) the germination rate was reduced to 30% for NaCl and to 10% for both KCl and MgCl_2_ (Fig. 4).

### Screening the mutant population to identify salt-tolerant lines

**Fig. 5:**
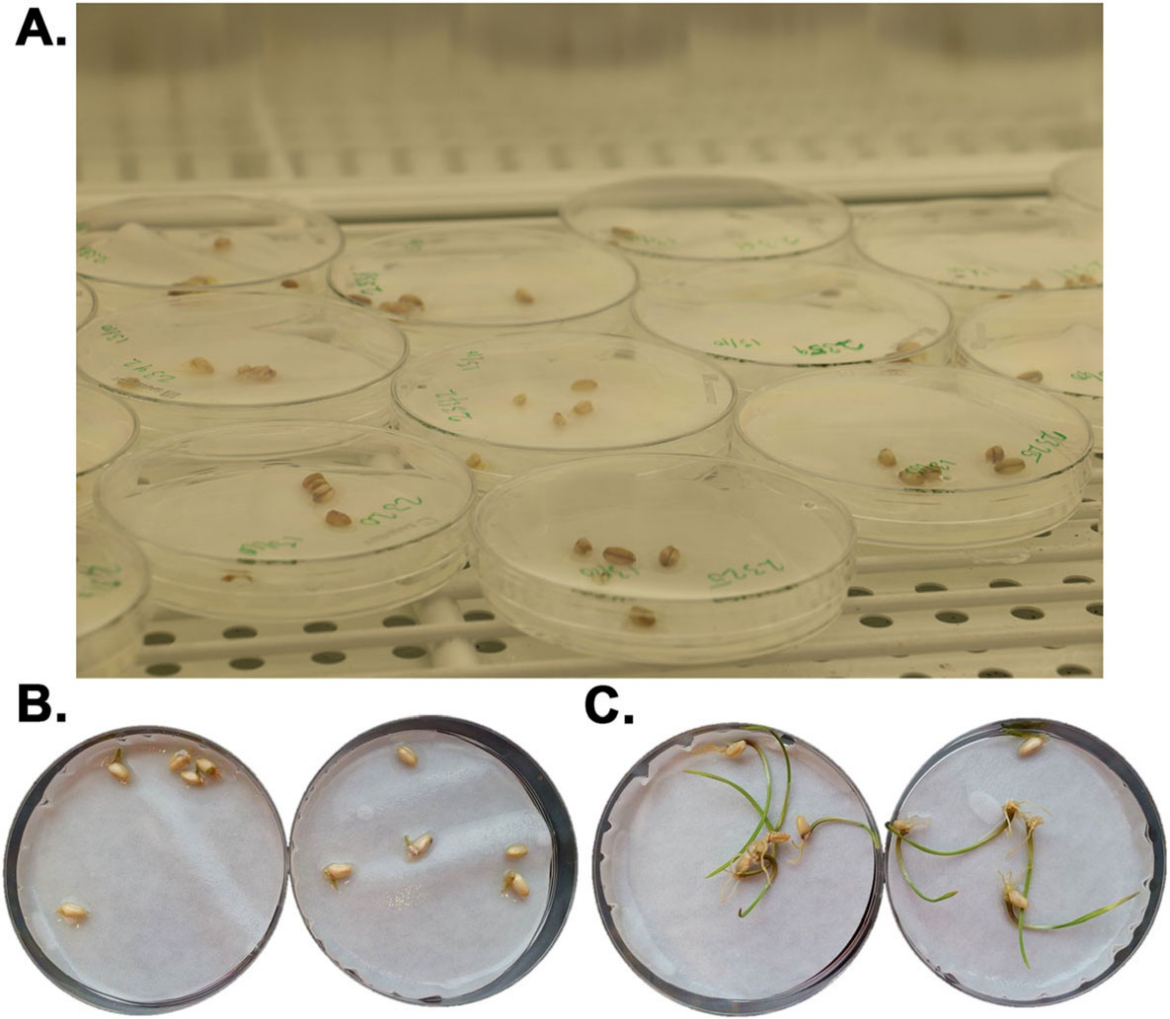
Screening for identification of salt-tolerant lines. **a** Ten seeds from each individual line were placed in two Petri dishes and grown on high salt (200 mM NaCl) at 22 °C and 240 μmol/m^2^/s light. After 7 days they were scored for germination. **b** Germination of BARI Gom-25 compared with **c** a strongly salt-tolerant mutagenized line with high germination rate at 200 mM NaCl

Based on the results of the Bari Gom-25 salt test our aim was to identify mutant lines giving at least 70% germination at 200 mM NaCl. We decided to use NaCl as the salt for this test as it is the most common saline stressor in soil. In addition to the Bari Gom-25 control, 1676 individual M4 generation lines were screened. Ten seeds were used for each mutated line and they were incubated either in Petri dishes with a soaked filter paper or in agar plates. From the 1676 lines tested, 70 lines germinated on the Petri dishes at a rate of 70% or higher (Fig. 5). These lines were then further propagated both in the greenhouse and in the field up to the current M8 generation.

### Root and shoot screening

**Fig. 6:**
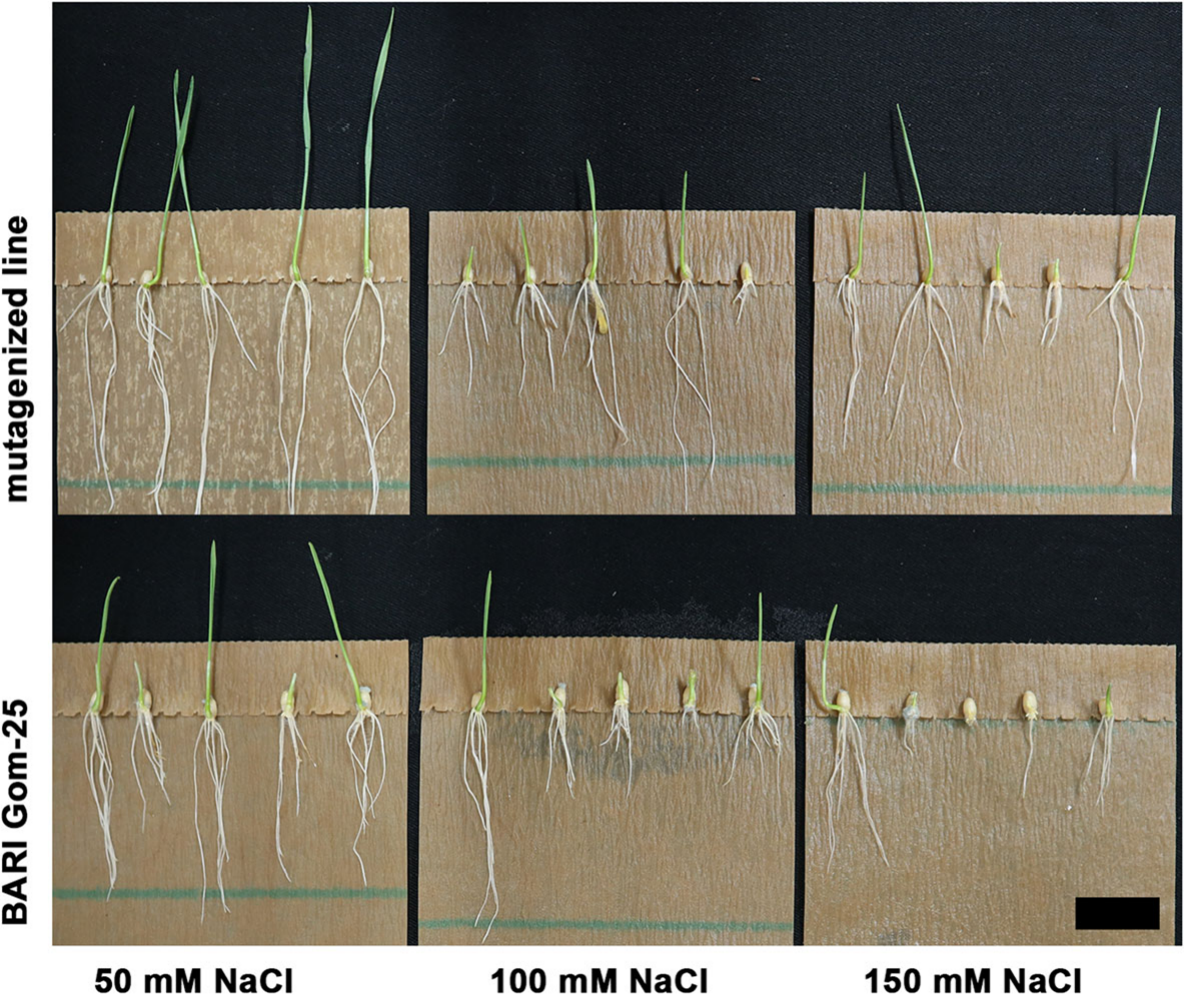
Root pouch test. Mutagenized wheat lines and BARI Gom-25 were placed in root pouches soaked with different concentration of NaCl. They were grown for 7 days in a growth chamber at 22 °C and 240 μmol/m^2^/s light. Top panel shows a representative mutagenized line and lower panel shows the control line BARI Gom-25. Black bar = 3 cm

**Fig. 7:**
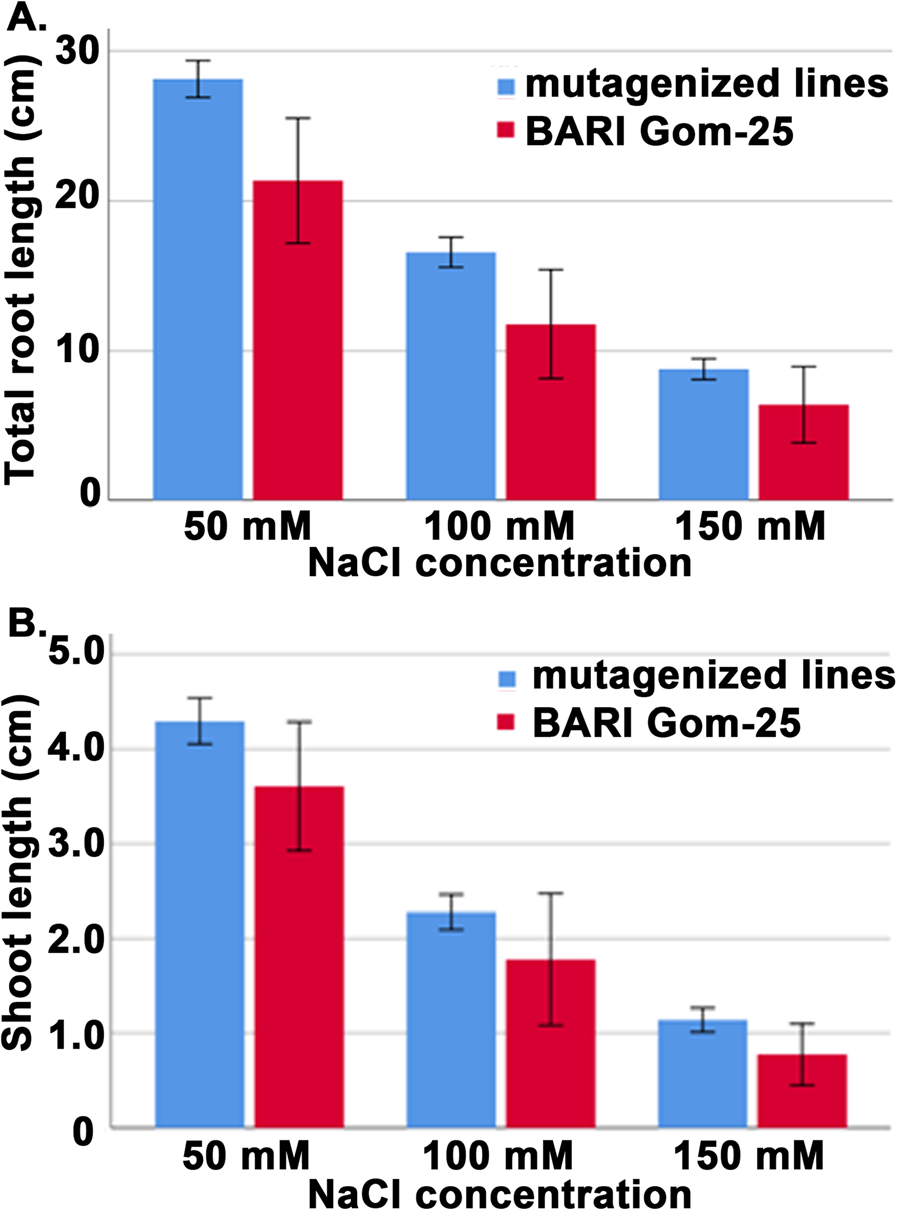
Root and shoot development during salt stress. Variation in root development (upper panel) and shoot development (lower panel) among mutagenized lines and BARI Gom-25 after treatment with different salt concentrations, using a root pouch. Error bars: 95% CI. Blue, mutagenized lines; red, BARI Gom-25

Root and shoots from the seed germination pouch assay were measured with a ruler and averages were calculated (Fig. 6). Then the 70 selected mutagenized lines were averaged as one group and compared to BARI Gom-25 as one group. This gave a better representation of the mean root and shoot development at the three salt concentrations tested (Table 1 and Figs. 6, 7). For the mutagenized population the root lengths showed a statistically significant increase in length at 50 mM and 100 mM NaCl compared to the BARI Gom-25 control. The same tendency could also be observed with 150 mM NaCl (Fig. 6). At 50 mM the average root length in the 70 mutants was 28.13 (±11.39) cm compared to 21.36 (±10.14) cm for BARI Gom-25, at 100 mM it was 16.57 (±9.49) cm compared to 11.77 (±8.78) cm, and at 150 mM it was 8.77 (±6.62) cm compared to 6.39 (±6.12) cm for BARI Gom-25 (Table 1, Fig. 6). The 70 mutagenized lines also showed better shoot development in all three salt concentrations, with an average shoot length at 50 mM NaCl of 4.29 (±2.23) cm compared to 3.60 (±1.65) cm for BARI Gom-25, at 100 mM NaCl 2.28 (±1.74) cm compared to 1.78 (±1.69) cm, and at 150 mM NaCl 1.14 (±1.21) cm compared to 0.77 (±0.79) cm for BARI Gom-25 (Table 1, Fig. 6). Finally, total mean root development (cm) per plant in all three different salt conditions was determined. These data showed that 51 out of the 70 mutated lines had better root development (measured as length and number of roots) than BARI Gom-25.

**Table 1:** Comparison of root and shoot development between the total amount of mutagenized lines and BARI Gom-25 for three different salt concentrations applied to root pouch assays

NaCl conc	Group	Mean root (cm)	SD root	Sign. (t-test)	Mean shoot (cm)	SD root	Sign. (t-test)
50 mM	Mut	28.13	11.39	0.004*	4.294	2.232	0.06
–	BARI Gom-25	21.36	10.14	–	3.606	1.645	–
100 mM	Mut	16.57	9.59	0.015*	2.278	1.743	0.166
–	BARI Gom-25	11.77	8.78	–	1.778	1.686	–
150 mM	Mut	8.77	6.616	0.082	1.138	1.208	0.04*
–	BARI Gom-25	6.39	6.173	–	0.773	0.790	–

*Significant difference *p* < 0,05

### Field trial

**Fig. 8:**
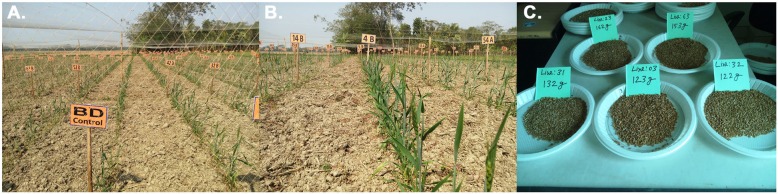
Field trials in coastal area of Kalapara, Patukhali (implemented by ICCO). The 70 best salt-tolerant lines and BARI Gom-25 were sown. **a, b** Each line was divided into three replicates and sown at three different places in the field. Tolerant lines showed better germination and growth vs. control. **c** One parameter (seeds harvested) analyzed

A field trial in a saline soil in southern Bangladesh was performed to test the 70 mutagenized lines identified in the germination screens and root tests. As a control, BARI Gom-25 was used. Two different seed lots were tested, one produced from mutagenized and BARI Gom-25 plants propagated in Sweden and one seed lot of BARI Gom-25 propagated in Bangladesh. This was done in order to assess possible differences in seed quality at the time of sowing due to previous seed adaptation. All seeds were planted in early December 2017. Seeds were sown in a row system in a north-south direction and 99 seeds were planted for most of the 70 lines. Each of the seed lots from these 70 lines was divided into three replicates and sown in several locations in the field to eliminate effects of local soil differences on the performance of each line (Fig. 8). The control BARI Gom-25 seeds from Sweden and Bangladesh were sown randomly among the 70 mutagenized lines to avoid local effects when comparing performance. The salinity at the start of the experiment was approximately 7.2 dS/m.

In January 2018, the germination rate of all lines was recorded and it was clear that all 70 mutagenized lines germinated better than either of the two different control BARI Gom-25 lines. The mutagenized lines had a germination rate ranging from 54.55 to 90.91%, while the BARI Gom-25 Bangladeshi seeds had 46.62%, and the BARI Gom-25 Swedish seeds had 15.15%. Furthermore, after two weeks of growth the 70 mutagenized lines were ranked at positions 1–67 for plant height, with the top mutagenized line showing an average height of 20.75 cm, while the BARI Gom-25 (Bangladesh) plants had an average height of only 5.51 cm and the BARI Gom-25 (Swedish) plants had an average height of 15.24 cm and were ranked at position 68. During the final stages of growth the soil salinity level had reached approximately 9.8 dS/m. By then the average plant height for BARI Gom-25 (Bangladesh and Sweden) was 63.50 cm and 60.95 respectively, while the best mutant lines had reached 71.12 cm, and 36 of the mutagenized lines had a height greater than or equal to that of BARI Gom-25. Total grain weights per total number of seeds germinated were also determined. This showed that the best mutagenized line produced 2.17 g/germinated seed compared to BARI Gom-25 with 1.87 g/germinated seed (Swedish seeds) and 1.12 g/germinated seed (Bangladeshi seeds). BARI Gom-25 (Sweden) produced 28 g of total grain weight from all the 99 seeds sown. Only 5 of the 70 mutagenized lines had lower values, probably due to fungal and/or insect infection. All lines from the mutagenized populations were found to be in the top category for single grain weight, germination rate and both combined. The controls were both found as outliers in the PCA plot.

**Fig. 9:**
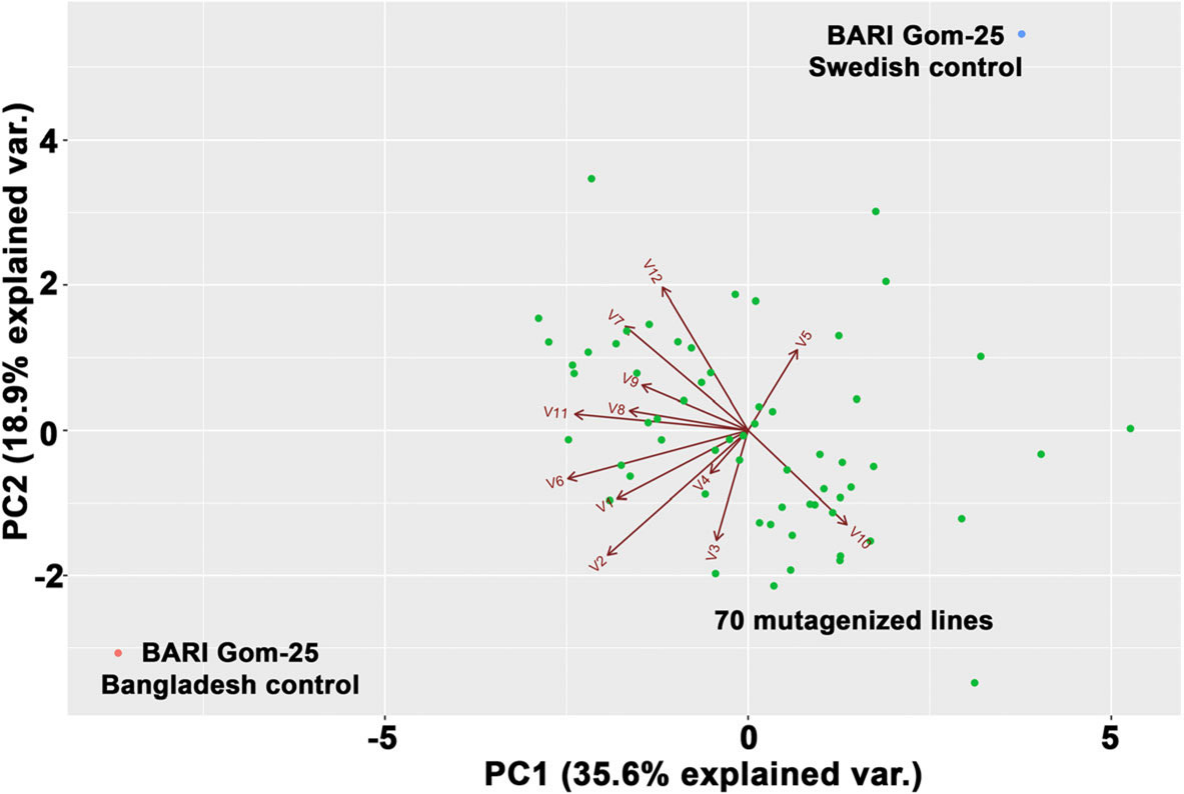
PCA plot of the field results from Bangladesh. Green dots represent the 70 mutagenized lines, the blue and red dots represent BARI Gom-25 controls cultivated in Sweden and Bangladesh respectively. Variables used for the PCA plot analysis were: V1, total seed sown; V2, total number of seeds germinating; V3, germination percentage; V4, average height; V5, position based on germination rate; V6, number of tillers harvested; V7, tillering percentage; V8, average length of spikelet; V9, number of kernels per spike; V10, fungal infection; V11, total grain weight; and V12, production per germinated seed

In addition, parameters such as amount of tillers, spikelet per tiller, length of spikelet, kernels per spike, fungal infection and insect infestation were measured, and here again the mutagenized population coped better with the saline conditions compared to BARI Gom-25. When combining all these parameters in a PCA analysis it was observed that the mutagenized lines clustered together whereas the BARI Gom-25 controls were outliers (Fig. 9). To select lines for further analysis and breeding purposes, a cut-off was applied for each parameter: germination rate of at least 60%, harvested tillers/germinated seed being at least 1, spike length over 2.54 cm, a minimum of 30 kernels per spike, less than 30% fungal infection, below 20% insect infestation, and minimum 1 in production per germinated seed. Based on these strict cut-off values, 17 OA-lines were retained for future analysis. Note that BARI Gom-25 did not meet the cut-off criteria.

### DNA preparation

Two of the mutagenized lines, OA70, and OA42, were chosen based on germination data and field results from Bangladesh. Genomic DNA (gDNA) was extracted from these lines as well as from the control variety BARI Gom-25. Since the aim was to isolate high molecular weight DNA, a CTAB-based protocol was used. For the PFGE, gDNA with molecular weights of around 50–90 kb were obtained. High molecular weight gDNA is important when using third-generation sequencing with reads longer than 10 kb, since it improves the assembly of complex genomes like that of wheat [31]. gDNA was extracted in duplicate from OA70 and OA42 and in triplicate from BARI Gom-25 and sequenced by the National Genomics Institute (NGI), Sweden. Whole-genome sequencing of OA70, OA42 and Gom24 generated a total of 4216.87 million reads with a length of 151 bp, and a total of 74,722, 100,105 and 114,210 million bp per genome respectively. The reads resulting from sequencing were of good quality as approximately 89% of all reads from all samples were above the Q30% cut off value.

### Mapping of the reads

The number of read pairs for BARI Gom-25, OA70, and OA42 were 747.03, 478.75 and 430.43 million reads respectively. The trimmed BARI Gom-25, OA70, and OA42 reads were aligned to the Chinese Spring Wheat reference genome using Bowtie2. The total numbers of reads for the two mutant samples after trimming and removing unmapped and multi-mapped reads were 370.63 and 334.23 million reads respectively. The alignment statistics showed that 98.67, 98.3 and 97.52% of the paired end reads were mapped to the reference genome for wild type, OA70 and OA42 respectively.

### EMS induced SNPs

**Fig. 10:**
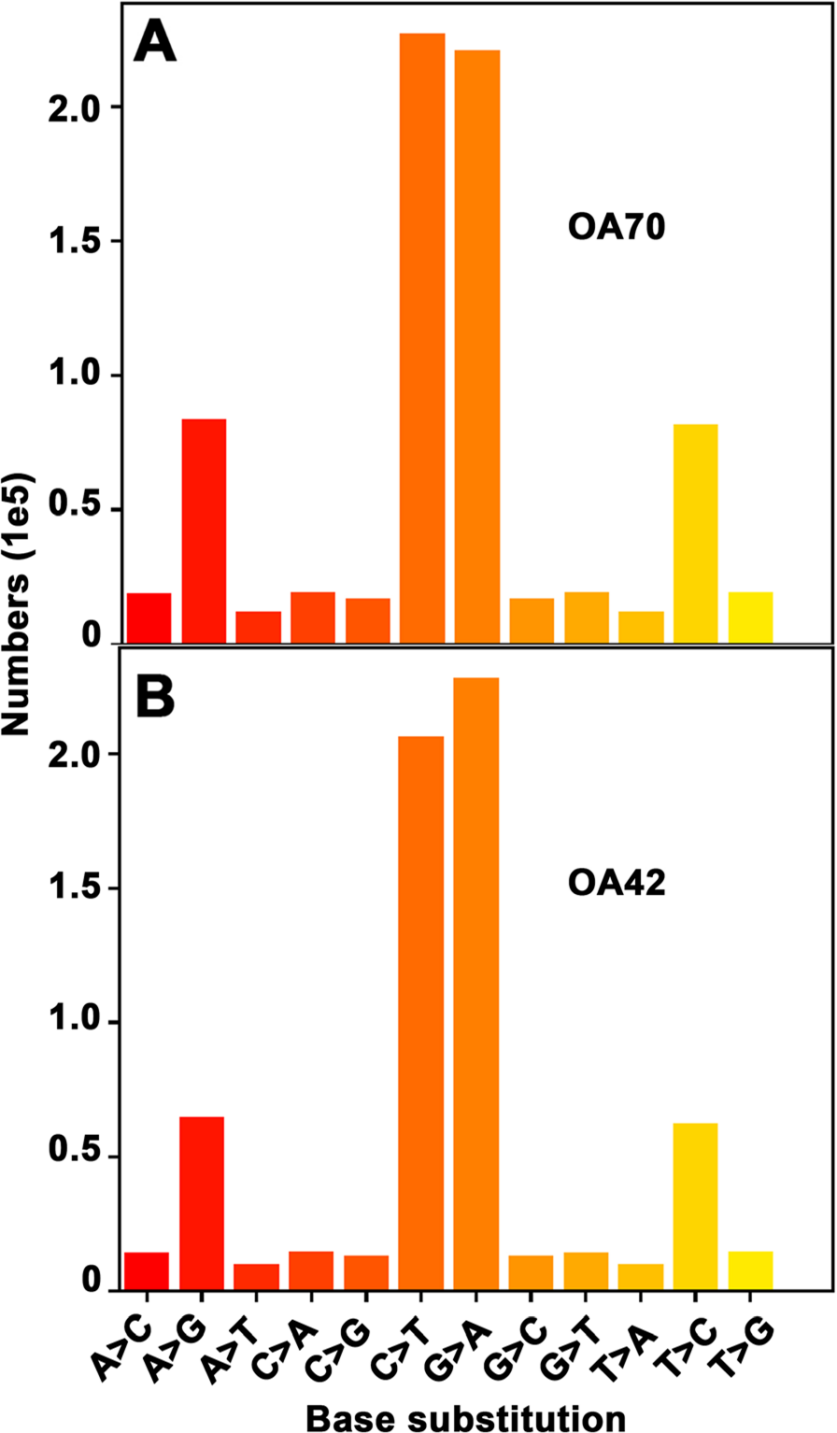
Characterization of EMS induced SNPs. The number of base substitution is shown for the mutagenized lines OA70 (**a**) and OA42 (**b**). The highest rate of base substitutions was observed for C to T and G to A

SNPs for BARI Gom-25, OA70, and OA42 were called against the Chinese Spring Wheat reference genome. Those SNPs common to the two mutants and BARI Gom-25 were removed, so that we could assume that the remaining SNPs in the two mutant samples would be EMS derived mutations compared to BARI Gom-25. After filtering, based on SAMtools, a total of 622,449 and 597,699 SNPs were identified for OA70 and OA42 respectively. Similarly, filtering the SNPs called using FreeBayes, a total of 768,954 and 683,201 were discovered for OA70 and OA42 respectively. Among the base changes in OA70, 82% were transitions and 18% were transversions. The numbers of transitions and transversions determined using SAMtools and FreeBayes are listed in Table 2. The most frequent transitions (Fig. 10) observed were G- > A (220536), C- > T (227030), T- > C (81548) and A- > G (83720). The transversions (Fig. 10A) consisted of C- > A (19003), G- > T (19037), A- > C (18810), T- > G (19006), G- > C (16774), C- > G (16967), T- > A (12201) and A- > T (12272).

**Table 2:** The number of transitions and transversions observed for the mutagenized lines using SAMtools and Freebayes

Type of substitution	Base to base conversion	OA70		OA42	
		SAMtools	FreeBayes	SAMtools	FreeBayes
Transition	G → A	219,110	220,536	250,786	228,122
	C → T	227,802	227,030	221,062	206,493
	T → C	46,420	81,548	33,158	64,847
	A → G	46,417	83,720	33,168	62,467
Transversion	C → A	12,932	19,003	9454	14,581
	G → T	12,991	19,037	9022	14,200
	A → C	10,707	18,810	7492	14,205
	T → G	10,612	19,006	7566	14,385
	G → C	10,160	16,774	7116	12,942
	C → G	10,053	16,967	6967	12,789
	T → A	7681	12,201	5916	9873
	A → T	7570	12,272	5994	9936

Similarly, for OA42, 84.6 and 15.4% of, respectively, transitions and transversions were observed (Fig. 10B). The order of frequency of transition types was as follows: G- > A (228122), C- > T (206493), A- > G (64847), T- > C (62467). Among the transversions, the order was G- > T (14200), C- > A (14581), A- > C (14205), T- > G (14385), G- > C (12942), C- > G (12789), T- > A (9873), A- > T (9936) (Fig. 10B).

**Fig. 11:**
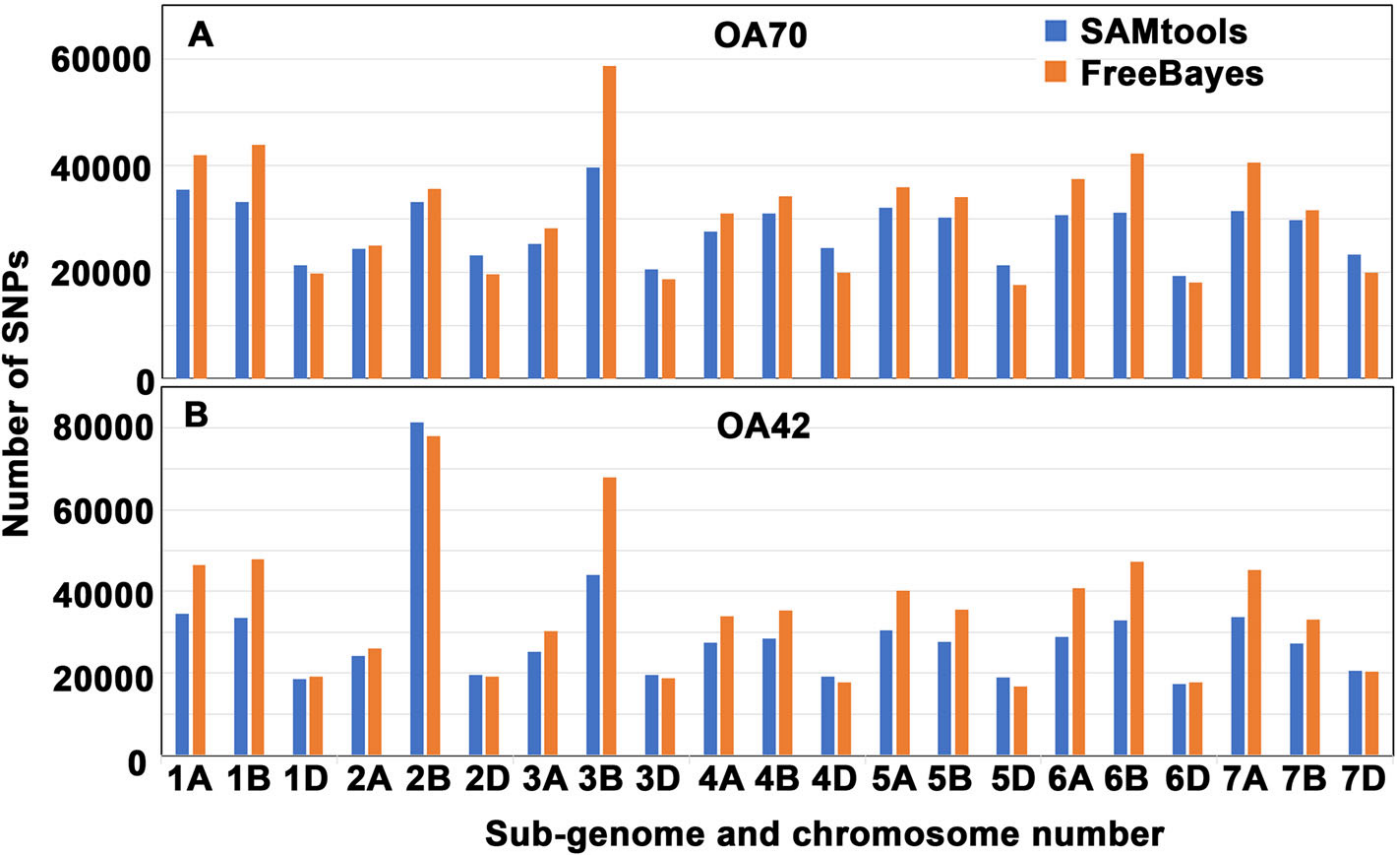
Distribution of SNPs in the sub-genomes of wheat. **a** Distribution of SNPs identified in the genome of OA70. SNPs identified using SAMtools and FreeBayes are coloured in blue and orange respectively. **b** Distribution of SNPs identified in the genome of OA42. SNPs identified using SAMtools and FreeBayes are coloured in blue and orange respectively. The three sub-genomes and the chromosome number are shown on the X axis. The number of SNPs is shown on the Y axis

OA70 exhibited greater sequence variation than OA42 when compared to the BARI Gom-25 reference genome. The genome-wide mutation rate for OA70 was one change per 18,918 bases, while it was one change per 21,292 bases in OA42. Among the three sub-genomes, A, B and D, the D genome was observed to have the lowest number of mutations in both the mutant lines. The genome-wide mutation rates for the A, B and D genomes in OA70 were 1 change per 18,765, 15,009 and 30,513 bases respectively. In OA42, the mutation rates for the A, B and D sub-genomes were one change per 20,514, 18,474 and 29,521 bases respectively. Among the 7 chromosomes for the three genomes, in OA70, the largest numbers of SNPs were observed in chr 1A, chr 2B and chr 7D (Fig. 11A). In OA42, the greatest numbers of SNPs was found in chr 1A, chr 3B and chr 7D (Fig. 11B).

Further examination of the three possible functional types identified 59.7 and 59.6% non-synonymous SNPs, 2.1 and 2.6% nonsense SNPs, and 38.1 and 37.7% of synonymous SNPs in OA70 and OA42 respectively. The non-synonymous/synonymous ratio was 1.5% for both OA70 and OA42.

## Discussion

Bangladesh is among the countries facing the maximum threat from climate change and it is also a country suffering from poverty. It is expected that both salinity and drought conditions will get worse. Finding new approaches to develop plant varieties that will sustain food production and improve food security is therefore imperative. Our idea for increasing agricultural productivity and reducing poverty by making unproductive land productive in a sustainable way is a step in this direction. Our aim is to do this by providing smallholder farmers in developing countries with affordable seeds.

Approximately 30% of the cultivatable land in Bangladesh is in the coastal area. About 1 million ha (corresponding to ca 40% of the cultivated area in Sweden) out of the ca. 2.9 million ha of coastal and offshore lands are affected by salinity. Large areas of the land therefore remain fallow in the dry season (January–May) due to excessive salinity levels. Crop production in the salt-affected areas in the coastal regions differs considerably from that in non-saline areas and with the increase of salinity in some areas due to saline water incursion, normal crop production is at risk. In these areas low yields and production levels will affect people’s livelihood even more than in other parts of the country where better-adapted modern agriculture technologies are used. At the same time food demand in the coastal area is increasing with the growing human population. Although wheat has good economic potential in the coastal belt, a number of technical and biological constraints need to be solved in order to explore its full potential. By taking advantage of modern molecular plant breeding activities to develop a wheat line with high salt-tolerance, land areas in the southern part of Bangladesh that suffer from high salinity could be better utilized.

Wheat crossbreeding approaches are continuously making impacts on food security world- wide, and this includes addressing salt tolerance. The Commonwealth Scientific and Industrial Research Organisation (CSIRO) identified genes involved in sodium exclusion (Nax1 and Nax2) that have been introduced into various crossing programs [32]. However, developing a new salt-tolerant variety based on introgression of genes from less domesticated lines is still a process whose precision is low and that takes a long time (ca 10–15 years). On the other hand, the process can be accelerated by an alternative approach based on increasing the variation in already domesticated populations and using such lines as a starting point (gene source) in a breeding program combined with selection using molecular markers.

By titrating to an optimal EMS concentration (1%, Fig. 1), a mutagenized wheat population were developed using a local Bangladeshi variety denoted BARI Gom-25. This variety was chosen because it was the most salt-tolerant Bangladeshi local variety at the start of this project (it still is), being classified as moderately salt-tolerant (~ 8–10 dS/m) in addition to being heat-tolerant [30]. Using 1% EMS solution to treat BARI Gom-25 reduced the germination rate by ca 80% and the survival rate for the M1 generation was 8.96%. This is in line with the initial titration curve that was constructed in order to find the optimal mutation conditions (Fig. 1). In total 2674 M1 lines were obtained and used to form a population of mutagenized lines. A larger population would give a higher total number of mutations, but also be more difficult to handle logistically, time wise, cost wise and technically. However, in a smaller population the mutation frequency needs to be high enough that, at the whole-population level, it creates mutations in every gene of the genome [25, 29].

Since EMS mutagenesis is random, every line in a mutated population will carry a different set of mutations. In the mutagenized BARI Gom-25 population, theoretically all genes have been mutated based on the calculated mutation frequency. A non-synonymous amino acid substitution may have an effect on enzyme activity, and a mutation upstream of a gene can, for example, affect regulatory regions, modify gene expression, or deregulate micro RNA. This means that all possible traits are theoretically present in the population, and at a later time they may also be designed by specific crossing using molecular markers for specific EMS mutations in specific mutant lines to ensure that homoeologous gene knock-outs are ‘stacked’. By designing a germination assay for high-salt tolerance (200 mM, corresponding to ca 20 dS/m) and by screening a population of 1676 different unique lines in the M4 generation, we identified 70 salt-tolerant lines with germination rates of at least 70% (Fig. 5). These lines thus considerably outperformed the BARI Gom-25 control.

In addition to the germination test, the 70 selected lines were further analyzed for root and shoot development at different NaCl concentrations, since previous studies have shown that root surface area and root hair decrease with increasing amounts of NaCl [33]. Thus a better ability to sustain root length in the mutagenized lines compared to BARI Gom-25 was considered to be a good indicator of salt tolerance. The observations made showed that the mutagenized lines had significantly longer roots at 50 mM and 100 mM NaCl and also a clear tendency towards longer roots at 150 mM (Fig. 7, Table 1). Moreover, shoot development was better at 50 mM and 100 mM NaCl for the mutagenized lines compared to BARI Gom-25 and significantly better at 150 mM (Fig. 7, Table 1). We did not include 200 mM as we wanted to remain within the range of what could be expected in the forthcoming field studies in Bangladesh (approximately 5–15 ds/m, equal to 50–150 mM). Based on the laboratory studies performed these 70 mutagenized lines were therefore selected for field trials in Bangladesh where they would be grown under more realistic conditions in order to compare the results to those of the laboratory experiments.

In December 2017 a field trial was set up using the 70 mutagenized lines previously selected, now at the M7 generation. The location chosen was Kalapara in the Patukhali district in Barisal in the southern part of Bangladesh. The trial was done in cooperation with a local farmer assigned via the Dutch NGO Interchurch Organization for Development Cooperation (ICCO). The soil salinity level was 7.2 dS/m at the sowing stage and it reached 9.8 dS/m before harvest. The seeds were sown in triplicate with a total of 99 seeds completely randomized on the field and with BARI Gom-25 as a control at 47 different positions throughout the field. The field plot was protected from feeding animals using a fence. First the germination rates of the sown seeds were determined. It was found that the mutagenized lines germinated significantly better than the BARI Gom-25 control. Since none of the seeds from the mutagenized lines had been propagated in Bangladesh, and thus they were not adapted to local conditions, this was somewhat unexpected. This was especially the case since a comparison between locally adapted BARI Gom-25 seeds and seeds propagated in Sweden showed that the adaptation effect was a major factor. The locally adapted BARI Gom-25 seeds had a germination rate of approximately 45%, but for the non-adapted BARI Gom-25 it was only ca 15%. In contrast, the best mutagenized line had a germination rate of more than 90%, despite the negative adaptation effect.

Several other parameters were used to monitor the plants throughout the growing season and the results showed that the mutagenized lines had an overwhelming advantage compared to the control. For example, 33 mutagenized lines produced more kernels per spike, 34 showed more tillers per plant, and the production per germinated seed for the best line was almost double compared to the BARI Gom-25 control (locally adapted). From a PCA plot it became obvious that BARI Gom-25 appeared as an outlier among the lines tested, in line with observations in the previous laboratory tests. In addition, when applying a cut-off filter to select the very best lines for future experiments, 17 lines appeared in the short-list and BARI Gom-25 was not among them. This gives excellent indications about the future capacity of the mutated population in general and as a starting point from which to develop salt-tolerant lines in particular.

The frequency of EMS mutations is known to be higher in polyploid species than in diploid species [34]. In the DNA sequences of the two EMS-mutagenized wheat lines, 71% of the mutations were G/C to A/T transitions, thus confirming that the mutations detected relative to the BARI Gom-25 reference were of the type expected for EMS treatment. A total of 768,954 and 683,201 SNPs were identified in the two mutant lines (OA70 and OA42), and they were randomly distributed throughout the three sub-genomes (A, B and D) of the wheat genome. OA70 had more mutations than OA42. The mutation rate for the OA70 and OA42 lines analyzed in this study were 1 in 18,918 and 21,292 bases respectively. The highest and lowest number of mutations was observed in the B and D sub-genomes respectively, for both the mutant lines. It has been reported that the number of polymorphisms in a mutagenized genome may scale with overall genome size [35]. Among the three sub-genomes of wheat, the order of genome size is B (5.180 Gb) > A (4.935 Gb) > D (3.951 Gb) [36]. This may explain why the two EMS mutants accumulated most mutations in the B genome and fewest in the D genome.

The predominant mutations in the two mutant lines were C:G > T:A changes, which are known to result from the alkylation of guanine residues. A:T > G:C transitions were observed to be more frequent compared to other substitutions. A similar observation has been reported previously in wheat [37–39]. Among the transversion types, the number of G to T (OA70) and C to A (OA42) transversions was the greatest, whereas the frequency of T to A transversions was the lowest in both the mutant lines. Low numbers of C:G > G:C transversions among EMS induced mutations have been reported earlier [40, 41]. Though these non-canonical mutations can be thought of as false positives, the occurrences observed are consistent with those reported in other studies on wheat [37, 38], tomato [41], pumpkin [42], and rice [43], suggesting that these changes are produced by EMS through unknown mechanisms.

EMS is one of the most effective ways of inducing mutations in a genome (in coding and non-coding regions). A non-synonymous SNP can result in a change in an amino acid in the protein sequence that can affect the functionality of the protein to varying extents. For both the mutants, the frequency of non-synonymous mutations (59%) was greater than that of synonymous mutations (38%). It will be necessary to analyze the impact of non-synonymous mutations on proteins with the aid of bioinformatics tools. Though the numbers of synonymous codon changes are smaller than those of non-synonymous mutations and such changes do not bring about any amino acid alterations, these changes can result in alterations in mRNA stability and ribosomal translation rates, and have many other effects [44].

## Conclusion

The major aim of this project was to develop systems for turning saline soil into productive soil in order to fight poverty and hunger. We chose Bangladesh as the first country in which to show proof of concept. With a population of 167 million people, Bangladesh is the world’s eighth most populated country. Starting from the well-adapted local Bangladesh wheat variety BARI Gom-25, we increased its genetic variation by EMS treatment. From the mutated BARI Gom-25 population we screened 1676 lines in the M4 generation for increased salt tolerance in a laboratory assay and identified 70 lines with high salt tolerance (20 dS/m) using a germination assay.

During the winter season (2017/2018) a field trial on saline land (7–10 dS/m) in Southern Bangladesh (Barisal) was performed with these 70 lines (M7 generation). The trial showed very promising results; all lines tested, including BARI Gom-25 as control, were ranked in a blind test by a local farmer and the 70 mutated lines were ranked in the first 70 places in germination tests. The plan is to repeat and scale up these field trials in Bangladesh and in the future to introduce the very best salt-tolerant lines for (DUS) testing so that they may become available to farmers in the future.

It is advantageous to start from a locally adapted variety, not only for obvious agricultural reasons, but also since such varieties will be more likely to gain acceptance among both growers and consumers. However this does not exclude the possibility that our mutated population can be used to produce molecular markers for salt tolerance; these will represent a very valuable breeding tool that will greatly facility introgression of salt tolerance into other wheat varieties around the world. This is of particular significance when taking into account the widespread legislation against GMO and genome editing around the world, which means that it is more advantageous to start from chemical mutations and introduce these into any wheat variety by classical crosses once they have been identified in mutant lines by sequencing. In addition, lines with not only salt tolerance, but also other quality characters such as high protein content and pathogen resistance could be selected from the population, to produce lines that would no doubt command a great value on the market.

## Methods

### Plant material

At the start of the project 2012 the local Bangladeshi wheat variety BARI Gom-25 was selected and obtained from the Bangladesh Agriculture Research Institute (BARI); it is both heat-tolerant and moderately salt-tolerant (10 dS/m). It gives good yields of around 3600–4600 kg/ha when sown under optimal conditions, and is resistant to leaf rust and bipolaris leaf blight [30, 45] (http://dhcrop.bsmrau.net/bari-gom-25/) (http://wheatatlas.org/country/varieties/BGD/0).

### Generating an EMS mutation population

BARI Gom-25 seeds were treated with different concentrations of EMS and grown in a greenhouse under optimized growth conditions for 15 days to verify the optimal EMS concentration as previously described [25]. It was found that 1% EMS killed ca 80% of all seeds, which is the level at which the maximum number of mutations was introduced in oat. To generate the M1 mutant population, approximately 30,000 seeds, divided into 200 seeds/tube, were treated with 1% EMS. Each lot of 200 seeds was then planted in 10 different pots with 20 seeds in each pot. Plants from the seeds that survived the EMS treatment and germinated were harvested, threshed and one seed from each surviving plant was replanted for further propagation. By the 4th generation 1676 individual lines, each with a unique combination of mutations, remained and were used for screening for salt tolerance.

### Screening and identification of salt-tolerant EMS mutated lines

An assay was developed to determine the germination frequency of BARI Gom-25 and different lines from the mutant population on saline media. Initially the sensitivity of BARI Gom-25 towards MgCl_2_, NaCl and KCl was determined by testing a concentration range at intervals of 50 mM from 0 to 350 mM for each salt. In each test, five seeds from each line were used and the seeds were placed on a filter paper soaked with 5 ml of the specific salt concentration. Plates were incubated using a light intensity of 240 μmol/m^2^/s, 18 h light/6 h night photoperiod, at 21 °C. The germination frequency was determined after 7 days and the general appearance of the seedlings documented. Germination was considered to be positive if the seedling had manifested root growth and sprouted the initial stem. For the BARI Gom-25 control, approximately 20% of seeds germinated at 200 mM NaCl. This NaCl concentration was then used to screen the whole mutant population of 1676 wheat lines in the M4 generation. Due to the large number of lines a labor-, time- and cost-efficient screen was applied in order to test all lines within a feasible time. From each line, ten seeds were used and divided between two Petri dishes each containing a filter paper soaked with 5 ml of 200 mM NaCl and as a control two plates (also containing a total of ten seeds) with 0 mM NaCl were used. Germination was evaluated after 7 days as in the initial trial and a germination rate of 70% or higher was considered to indicate strong salt tolerance.

### Root and shoot screening

To further confirm the increased salt tolerance of the salt-tolerant lines identified, a root assay was developed using a seed germination pouch (Phyto AB). Using tweezers, seeds were placed with the embryo side down at specific positions in the pouch and 10 ml of a NaCl solution was poured into the pouch. Three different NaCl concentrations (50 mM, 100 mM, 150 mM) were used. Each pouch was placed in a vertical position under a light intensity of 240 μmol/m^2^/s at 22 °C with an 18 h light/6 h dark photoperiod. The pouches were incubated for 7 days before root lengths were measured and general root architecture documented.

### Field trials and principal component analysis

The salt-tolerant wheat lines identified were grown in a field of area ca 1100 m^2^ in Kalapara in the Patukhali district of Bangladesh together with the control variety BARI Gom-25. The field is a demo plot run by local resource farmers supervised by the Interchurch Organisation for Development Co-operation (ICCO Cooperation, Bangladesh) as part of their Salt Solution Project following national legislation. The land was ploughed to a depth of ca 12–15 cm with a power tiller, and 14 ditches were made for effective drainage. At the start of the experiment, the salinity of the soil was monitored and found to be around 7.2 dS/m. As the growing season continued and the soil dried out, the salinity increased, reaching ca 9.6 dS/m at the end of the season. For most of the 70 lines tested, 99 seeds were divided into three replicates of 33 seeds, each planted in randomized plots in the field, sown in a row system in the north-south direction. Plant to plant distance was 10 cm and row to row distance 1 m, with a total of 26 rows. The parameters that were scored during the experiment were germination rate, plant and spikelet height, number of kernels, number of tillers, total grain weight and production per germinated seeds (grain weight/total germinated seeds).

A principal component analysis (PCA) containing the following variables: total seed sown, total seeds germinated, germination percentage, average height (cm), germination rate, number of harvested tillers, tillering percentage (harvested tillers/germinated seed*100), average length of spikelet, number of kernels per spike, % plants with fungal infection, % plants with insect infestation, total grain weight (g), and production per germinated seed was then used to identify which of the different field parameters recorded made the greatest contribution to saline tolerance. For the analysis, the data input for PCA was organized as a matrix denoted X, which was composed of N and K dimensions, where N represents the number of salt-tolerant lines (observations) and K represents the number of field observations (variables). In the PCA, a principal component is defined, i.e. the K-dimensional space along which the variance of the data set is the greatest. The orientation of the model plane in the K-dimensional variable space is set by the loadings, quantifying the contribution of each of the original variables to the principal components. The principal components are the eigenvectors of the covariance matrix of the data matrix X and are thus orthogonal. The eigenvectors associated with the largest eigenvalues of the data correspond to the direction of the greatest variation in the data.

### DNA preparation

BARI Gom-25 and two mutant lines, OA70, and OA42, were used for extracting genomic DNA. Five plants from each mutant line were grown for 8 days in darkness at 20 °C, and etiolated seedlings were cut and pooled to produce one sample for each mutant line. The material was ground into fine powder using liquid nitrogen. DNA was extracted using a modified CTAB protocol [46, 47], in which 10 ml of CTAB buffer (Tris-HCl (pH 7.5), 25 mM EDTA, 1.5 M NaCl, 2% (w/v) CTAB, 0.3% (v/v) β-mercaptoethanol) was added to 1 g of finely powdered leaf material, and gently mixed every ten minutes for 40 min at 65 °C. Samples were centrifuged at 5000 g for 5 min, at 20 °C (without cooling the rotor to avoid precipitation of CTAB), to collect the supernatant. The same volume of chloroform: isoamyl alcohol 24:1 was added to the supernatant and the mixture was centrifuged at 5000 g, 5 min, at 20 °C. The supernatant was removed to a new tube, and 5 μl RNase (10 mg/ml) was added and the mixture incubated at 37 °C for 15 min with occasional swirling. An equal amount of chloroform: isoamyl alcohol 24:1 was added and the sample was centrifuged for 5 min, 5000 g, at 20 °C before the supernatant was slowly transferred to a new tube. A half volume of 5 M NaCl was added and swirled before adding three volumes of 95% EtOH chilled to − 20 °C and incubating 1 h at − 20 °C. The samples were collected and spun at 5000 g for 10 min. The pellet was washed with 5 ml 76% EtOH, 200 mM Na-Ac for 15 min, then 76% EtOH, 10 mM NH_4_-Ac for 5 min, and finally 75% EtOH for 2 min. After each washing step the sample was spun at 5000 g for 5 min. The pellet was air dried for 15 min at room temperature before being resuspended in 500 μl 50 mM Tris buffer overnight at 5 °C. The next day the sample was centrifuged at maximum speed and the DNA-containing supernatant was transferred to a fresh tube and stored at − 4 °C until required for use.

### DNA quality control

The DNA concentration was determined using a Qubit fluorometer (Fisher Scientific) and the purity was confirmed using a NanoDrop (Thermo Scientific). Pulsed Field Gel electrophoresis was used to verify DNA length according to electric charge and size [48].

### DNA sequencing and processing of the data

The library was constructed using Illumina’s TruSeq PCR-free library preparation kit with an insert size of 350 bp and a read length of 151 bp. The paired-end sequencing of the three samples, from wild-type BARI Gom-25 and the two OlsAro lines OA42 and OA70, was performed on an Illumina HiSeq X sequencing machine at SciLifeLab, Sweden, resulting in a total of 116,271 million base pairs. Potential PCR duplicates at rates of 6, 5.6 and 6.1% were found and subsequently excluded from the BARI Gom-25, OA70 and OA42 samples respectively. To ensure sequencing quality, all the sequences were subjected to quality control checks such as determining yield, sequence read quality and cross-sample contamination. The quality of sequences was also checked using FastQC [49]. The adapter sequences in the reads were trimmed before analysis according to the standard SciLifeLab procedure.

### Alignment of reads to reference genome

The recently annotated Chinese Spring wheat genome [50] was downloaded from Ensembl Plants (IWGSC RefSeq v1.0) and used as reference for aligning the reads. Bowtie2 (Version 2.3.3.1) [51] with the option “very-sensitive” was then used to align the Illumina data from BARI Gom-25, OA70, and OA42 to the Chinese Spring reference genome. Alignments were processed using SAMtools v.1.3.1 [52]. To eliminate reads that aligned to multiple sites due to the complexity of the genome, the multiply-mapped and unmapped reads were subsequently removed using SAMtools option. The successful removal of multi-mapped and unmapped reads was further verified using SAMtools flagstat. Further processing included converting Sequence Alignment Map (SAM) to Binary Alignment Map (BAM) format, sorting (based on coordinates), sub-selecting reads of mapping quality > 30 and merging the BAM files for each mutant replicate generated from different sequencing lanes. Mapping statistics for the three genomes were obtained using the SAMtools flagstat command.

### SNP calling

Two different variant callers, SAMtools and FreeBayes, were used to identify SNPs. The mpileup command in SAMtools was used for detecting SNPs from the sorted BAM files after removing duplicates using Picard tools (Version 2.1.1) (https://broadinstitute.github.io/picard/) with the parameters -u (to generate BCF output) -E (for extended Base Alignment Quality for greater sensitivity of local realignment around short indels) -C 50 (to adjust mapping quality) –BCF (to generate genotype likelihood in BCF format) and -f (the path to the reference Chinese Spring wheat genome). The BCFtools call command was used to call the SNPs. FreeBayes was run with parameters -C 2 (minimum alternate count), −F 0.050 (minimum alternate fraction) -q 20 (minimum base quality) -p (level of polyploidy). The SNPs were filtered using BCFtools and VCFtools v.0.1.13 [53] based on total depth (DP > 5) and quality score (QUAL > 20).

## Data Availability

The data that support the findings of this study are available from the corresponding author upon reasonable request.

## Abbreviations

BAM: Binary Alignment Map
BARI: Bangladesh Agriculture Research Institute
EMS: ethyl methanesulfonate
ICCO Cooperation, Bangladesh: Interchurch Organisation for Development Co-operation
PCA: principal component analysis
SAM: Sequence Alignment Map
